# Utility of cerebrospinal fluid liquid biopsy in distinguishing CNS lymphoma from cerebrospinal infectious/demyelinating diseases

**DOI:** 10.1002/cam4.6329

**Published:** 2023-07-27

**Authors:** Chisako Iriyama, Kenichiro Murate, Sachiko Iba, Akinao Okamoto, Naoe Goto, Hideyuki Yamamoto, Toshiharu Kato, Keichiro Mihara, Takahiko Miyama, Keiko Hattori, Ryoko Kajiya, Masataka Okamoto, Yasuaki Mizutani, Seiji Yamada, Tetsuya Tsukamoto, Yuichi Hirose, Tatsuro Mutoh, Hirohisa Watanabe, Akihiro Tomita

**Affiliations:** ^1^ Department of Hematology Fujita Health University School of Medicine Toyoake Japan; ^2^ Department of Neurology Fujita Health University School of Medicine Toyoake Japan; ^3^ International Center for Cell and Gene Therapy Fujita Health University Toyoake Japan; ^4^ Department of Hematology and Oncology Fujita Health University Okazaki Medical Center Okazaki Japan; ^5^ Department of Pathology Fujita Health University School of Medicine Toyoake Japan; ^6^ Department of Neurosurgery Fujita Health University School of Medicine Toyoake Japan

**Keywords:** *CD79B*, central nervous system lymphoma, genetic mutations, liquid biopsy, *MYD88*

## Abstract

**Background:**

Distinguishing between central nervous system lymphoma (CNSL) and CNS infectious and/or demyelinating diseases, although clinically important, is sometimes difficult even using imaging strategies and conventional cerebrospinal fluid (CSF) analyses. To determine whether detection of genetic mutations enables differentiation between these diseases and the early detection of CNSL, we performed mutational analysis using CSF liquid biopsy technique.

**Methods:**

In this study, we extracted cell‐free DNA from the CSF (CSF‐cfDNA) of CNSL (*N* = 10), CNS infectious disease (*N* = 10), and demyelinating disease (*N* = 10) patients, and performed quantitative mutational analysis by droplet‐digital PCR. Conventional analyses were also performed using peripheral blood and CSF to confirm the characteristics of each disease.

**Results:**

Blood hemoglobin and albumin levels were significantly lower in CNSL than CNS infectious and demyelinating diseases, CSF cell counts were significantly higher in infectious diseases than CNSL and demyelinating diseases, and CSF‐cfDNA concentrations were significantly higher in infectious diseases than CNSL and demyelinating diseases. Mutation analysis using CSF‐cfDNA detected *MYD88* ^L265P^ and *CD79* ^Y196^ mutations in 60% of CNSLs each, with either mutation detected in 80% of cases. Mutual existence of both mutations was identified in 40% of cases. These mutations were not detected in either infectious or demyelinating diseases, and the sensitivity and specificity of detecting either *MYD88/CD79B* mutations in CNSL were 80% and 100%, respectively. In the four cases biopsied, the median time from collecting CSF with the detected mutations to definitive diagnosis by conventional methods was 22.5 days (range, 18–93 days).

**Conclusions:**

These results suggest that mutation analysis using CSF‐cfDNA might be useful for differentiating CNSL from CNS infectious/demyelinating diseases and for early detection of CNSL, even in cases where brain biopsy is difficult to perform.

## Graphical abstract

Genetic analyses using cerebrospinal fluid cell‐free DNA (CSF‐cfDNA) (liquid biopsy) is a useful strategy for distinguishing central nervous system lymphoma (CNSL) from CNS infectious/demyelinating diseases. Genetic analysis using CSF‐cfDNA can be contribute to earlier detection of CNSL than conventional methods.

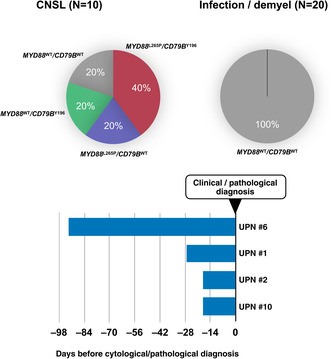

## 1 INTRODUCTION

Primary central nervous system lymphoma (PCNSL) is a rare subtype of extranodal lymphoma that primarily affects the cerebrum, brainstem, and spinal cord.^1^ Additionally, systemic malignant lymphomas, such as diffuse large B‐cell lymphoma (DLBCL), sometimes infiltrate the CNS during disease progression or relapse.^2^ While patients with PCNSL might exhibit symptoms such as headache, progressive sensory and/or motor involvement, behavioral changes, and impaired consciousness, these symptoms are also commonly seen in other CNS disorders, such as infectious/inflammatory brain diseases and brain tumors. Thus, obtaining a definitive diagnosis of PCNSL is often challenging, even with the use of conventional imaging technologies, such as computed tomography (CT) and magnetic resonance imaging (MRI), as well as biochemical and cytological analysis of cerebrospinal fluid (CSF).^3^

A definitive diagnosis of PCNSL is ideally made via a brain biopsy. However, this is an invasive procedure and might not be possible due to the location of the tumor or institutional limitations. Although a tentative diagnosis can be made by cytology and/or flow cytometry (FCM) analysis of tumor cells in CSF, in some cases, positive findings might not be obtained for several months, until the disease progresses sufficiently to be diagnosed by these analyses.^4^ Since a delay in diagnosis is associated with poorer prognosis and worse quality of life due to irreversible neurological effects, it is desirable to establish a methodology for the early detection and differentiation of PCNSL from other diseases.

Reportedly, approximately 90% of PCNSL cases are DLBCLs,^5^ and recent studies have shown an accumulation of mutations in genes such as *MYD88, CD79B, TBL1XR1, PIM1, IGLL5*, and *BTG1* in PCNSL cases.^6^, ^7^, ^8^, ^9^, ^10^, ^11^ In particular, *MYD88* ^L265P^ and *CD79B* ^Y196^ mutations have been reported in 27%–71% of cases, indicating their involvement in the pathogenesis of PCNSL. According to comprehensive genetic analysis, DLBCL cases harboring *MYD88* and *CD79B* mutations are classified as the MCD type^12^, ^13^ and the cluster 5 type,^14^ a characteristic subgroup with poor prognosis. *MYD88/CD79B* mutations are also observed in other subtypes of DLBCL, such as primary testicular DLBCL,^11^, ^15^ primary breast DLBCL,^16^, ^17^ primary cutaneous DLBCL leg‐type,^12^ intravascular large B cell lymphoma (IVLBCL),^18^ and malignant retinal lymphoma,^19^, ^20^ and these subtypes have a higher incidence of CNS infiltration than subtypes without *MYD88/CD79B* mutations.^6^ These genetic abnormalities have the potential to become interesting markers for disease detection and CNS invasion.^2^, ^6^

Liquid biopsy, a technique that utilizes cell‐free DNA (cfDNA) extracted from body fluids, such as peripheral blood plasma, is used to detect genetic mutations in tumor cells.^21^ This method is currently being utilized in translational research for genetic mutation analyses, and in the detection of minimal residual diseases in various fields, including DLBCL,^18^, ^22^, ^23^, ^24^, ^25^ Hodgkin lymphoma,^26^, ^27^ T‐cell lymphomas,^28^, ^29^ and IVLBCL.^18^ The use of liquid biopsy using CSF has also been studied in the context of PCNSL, in the hope that it might contribute to diagnosis of this condition.^4^, ^20^, ^30^, ^31^, ^32^, ^33^ Furthermore, recent reports suggest the potential advantage of liquid biopsy in CNSL in enabling earlier detection of tumors than conventional diagnostic strategies.^4^, ^32^

However, information on mutation status in benign diseases other than CNSL, which need to be distinguished clinically, still needs to be accumulated. In this study, we focused on CNSL and CNS infectious/demyelinating diseases and confirmed the benefits of liquid biopsy using CSF‐cfDNA by utilizing the droplet‐digital PCR (ddPCR) method, a relatively simple quantitative detection strategy that can potentially be utilized in a clinical setting. We also compared biochemical test values in peripheral blood and CSF, to further confirm the sensitivity and specificity of presence of *MYD88* ^L265P^/*CD79*B^Y196^ mutations in CNSL cases for differentiating CNSL from inflammatory/demyelinating diseases. In addition, whether genetic analysis using CSF‐cfDNA is more useful than conventional diagnostic strategies for the early detection of CNSL was confirmed even in the cases where brain biopsy is difficult to perform.

## 2 MATERIALS AND METHODS

### 2.1 Patients' characteristics

Patients diagnosed with malignant lymphoma with CNS involvement (CNSL; *N* = 10) (Table S1; Figure S1), infectious diseases of the CNS (*N* = 10), or demyelinating disease of the CNS (*N* = 10) at Fujita Health University Hospital between August 2018 and July 2021 were retrospectively included in the analysis (Tables 1 and 2). CNSL included primary CNSL (PCNSL, *N* = 3), recurrent disease with CNS involvement only (secondary CNSL, SCNSL, *N* = 3), and systemic lymphoma cases with CNS involvement (DLBCLc; *N* = 4). Infectious diseases included meningitis (*N* = 7), encephalitis (*N* = 2), and encephalomeningitis (*N* = 1). Demyelinating diseases included multiple sclerosis (MS; *N* = 8) and neuromyelitis optica spectrum disorder (NMOSD; *N* = 2). We utilized CSF samples collected during epidural anesthesia for CSF analysis in cases without CNS diseases as controls. Informed consent was obtained from all cases. This study was performed in accordance with the Declaration of Helsinki and the domestic ethical guidelines issued by the Ministry of Health, Labour and Welfare in Japan. This study was approved by the institutional review board of Fujita Health University (approval numbers, HG18‐017, HG20‐055, HG21‐028, and HG22‐006).

**TABLE 1:** Comparison of laboratory data for peripheral blood and cerebrospinal fluid in patients with CNSL, infectious diseases, and demyelinating diseases.

		CNSL	Infectious diseases	Demyelinated diseases	*p* value
		(*N* = 10)	(*N* = 10)	(*N* = 10)	
		Median (IQR)	Median (IQR)	Median (IQR)	
Age		68 (63–73)	47 (28–71)	46 (34–52)	0.0097*
Gender (Male%)		50	60	20	
PB					
WBC	/μL	5250 (4550–11,075)	9825 (5025–9825)	5450 (3850–6575)	*0.2832*
Lymphocyte	/μL	1231 (642–1518)	1145 (725–1377)	1117 (558–1678)	*0.9249*
Hb	g/dL	10.6 (9.9–12.1)	14.6 (13.0–16.9)	13.3 (12.2–13.9)	0.0004*
Plt	/μL	19.9 (14.4–24.2)	24.9 (19.9–28.8)	26.7 (22.3–30.5)	*0.0899*
TP	g/dL	6.2 (5.9–7.4)	7.2 (7.0–7.5)	6.8 (6.4–7.4)	*0.1059*
Alb	g/dL	3.6 (2.65–3.93)	4.3 (4.0–4.7)	4.2 (4.0–4.5)	0.0014*
LD	U/L	215 (167–427)	211 (189–261)	181 (139–205)	*0.1281*
CRP	mg/dL	0.90 (0.07–3.05)	0.07 (0.04–0.62)	0.05 (0.03–0.13)	*0.0677*
CSF					
Protein	mg/dL	60.5 (43.5–171.3)	94.5 (66.5–137.8)	40.0 (27.3–54.0)	0.0034*
Glucose	mg/dL	63.5 (53.75–73.0)	59.0 (45.5–67.5)	59.0 (55.0–79.3)	*0.5436*
Cell counts					
Total	/μL	5.5 (1.75–20.5)	236.0 (46.8–496.3)	2.5 (2.0–6.8)	0.0002*
MNC	/μL	5.0 (1.0–19.25)	126.0 (29.8–353.5)	1.5 (1.0–4.5)	0.0002*
PMN	/μL	1.0 (0–1.0)	13.5 (4.8–47.0)	1.0 (0–2.0)	<0.0001*
cfDNA concentration	ng/μL	11.50 (4.48–16.93)	59.48 (16.13–123.8)	5.825 (4.483–9.045)	0.0007*

*Note:* Asterisks indicate values that are significantly different. *p* Value is indicated in the italic character.

Abbreviations: Alb, albumin; cfDNA, cell‐free DNA; CNSL, central nerves system lymphoma; CRP, C‐reactive protein; CSF, cerebrospinal fluid; Hb, hemoglobin; LD, lactate dehydrogenase; MNC, mononuclear cells; PB, peripheral blood; Plt, platelet; PMN, polymorphonuclear cells; TP, total protein; WBC, white blood cells.

**TABLE 2:** CSF‐cfDNA concentration and variant allele frequency of genetic mutations in CSF‐cfDNA in patients with CNSL, infectious diseases, and demyelinating diseases.

UPN	Diseases groups	Diagnosis	Sex	Age	CSF cfDNA concentration [ng/mL]	CSF‐cfDNA		FFPE	
						*MYD88* (L265P) VAF %	*CD79B* (Y196) VAF %	*MYD88* (L265P) VAF %	*CD79B* (Y196) VAF %
1	CNSL	PCNSL	M	61	40.3	23	1.26 (Y196N)	N.A.	N.A.
2		PCNSL	F	73	5.9	38	11.5 (Y196N)	47.9	LOS
3		PCNSL	F	65	4.8	58.8	5.7 (Y196N)	N.A.	N.A.
4		SCNSL	F	72	3.5	LOS	LOS	N.A.	N.A.
5		SCNSL	M	73	11.3	29.3	LOS	N.A.	N.A.
6		SCNSL	F	53	2.44	LOS	1.49 (Y196H)	8.0	LOS
7		DLBCLc	M	66	11.7	34.4	LOS	N.A.	N.A.
8		DLBCLc	M	70	16.4	6.6	85.3 (Y196H)	N.A.	N.A.
9		DLBCLc	M	76	12.2	LOS	LOS	LOS	LOS
10		DLBCLc	F	63	18.5	LOS	1.18 (Y196N)	LOS	LOS
11	Infectious diseases	Meningitis	M	55	41.29	LOS	LOS		
12		Encepharomeningitis	M	72	187.39	LOS	LOS		
13		Meningitis (viral)	M	18	96.11	LOS	LOS		
14		Encephalitis (viral)	M	58	8.26	LOS	LOS		
15		Meningitis (viral)	F	31	135	LOS	LOS		
16		Meningitis (viral)	F	26	77.67	LOS	LOS		
17		Meningitis (viral)	M	38	120	LOS	LOS		
18		Meningitis (viral)	M	28	18.56	LOS	LOS		
19		Meningitis (viral)	F	71	8.85	LOS	LOS		
20		Meningitis (viral)	F	71	28.55	LOS	LOS		
21	Demyelinated diseases	MS	F	28	7.24	LOS	LOS		
22		MS	F	42	6.47	LOS	LOS		
23		MS	F	49	4.01	LOS	LOS		
24		MS	F	44	4.64	LOS	LOS		
25		MS	F	51	9.6	LOS	LOS		
26		MS	M	36	8.86	LOS	LOS		
27		NMOSD	F	54	9.93	LOS	LOS		
28		NMOSD	F	81	2.73	LOS	LOS		
29		MS	M	47	4.88	LOS	LOS		
30		MS	F	17	5.18	LOS	LOS		

*Note:* The shade columns indicate positive result for mutation.

Abbreviations: CNSL, central nerves system lymphoma; DLBCLc, diffuse large B cell lymphoma with CNS involvement; FFPE; formalin fixed paraffin embedded tissue sample; LOS; limit of sensitivity; MS, multiple sclerosis; N.A., not available; NMOSD, neuromyelitis optica spectrum disorders; PCNSL, primary CNSL; SCNSL, secondary CNSL; VAF, valiant allele frequency.

### 2.2 Sample preparation

CSF collected for the differential diagnosis of CNS diseases in a clinical setting was primarily used for cytological and biochemical examinations, and residual specimens were stocked at −30°C for further use. CSF samples from patients with infectious/demyelinating diseases were obtained during the acute phase of symptom onset. Cell‐free DNA in CSF (CSF‐cfDNA) was extracted from cryopreserved CSF specimens using the QIAamp Circulating Nucleic Acid Kit (QIAGEN) as previously reported^4^ and stored at −30°C for further analyses. In cases in which brain biopsies were performed, genomic DNA was extracted from formalin‐fixed paraffin‐embedded (FFPE) tissue samples by LSI Medience Corporation (Tokyo, Japan) or residual samples of cells used for FCM analysis. DNA concentration was measured using a Qubit 4 Fluorometer (Thermo Fisher Scientific) as described previously.^4^, ^18^ Blood tests were performed closer to the date of CSF collection, and the relevant data were extracted from the patients' electronic medical records and used in the analysis.

### 2.3 Mutational analyses

Mutational analyses for *MYD88* ^L265P^ and *CD79B* ^Y196^ were performed using ddPCR (Bio‐Rad Laboratories, Hercules, CA, USA) as indicated previously.^4^ Since CSF‐cfDNA concentrations are usually as low as 10 ng/mL (Table 2), ddPCR was performed using amplicons (1 × 10^−5^ ng/reaction), PCR fragments of the TIR domain of *MYD88* containing the *MYD88* ^L265P^ site and the immunoreceptor tyrosine‐based activation motif (ITAM) domain containing the *CD79B* ^Y196^ site amplified by using PCR primers as follows: MYD88‐TIR‐4U; 5′‐GTT AAC CCT GGG GTT GAA GAC TG‐3′, MYD*88*‐TIR‐4L; 5′‐TAC ATG GAC AGG CAG ACA GAT ACA C‐3′, CD79B‐ITAM‐U; 5′‐GAC ACT AAC ACT CTG ATC TCC‐3′, and CD79B‐ITAM‐L; 5′‐GAC CAC TTC ACT TCC CCT GT‐3′ (amplicon‐based ddPCR).^4^ Note that quantitativity of the valiant allele frequency has been confirmed by basic experiments using plasmid DNA, as reported previously.^4^ Based on the previous results of quantitation by titration^4^ and examination using CSF‐cfDNA specimens from non‐tumorigenic patients who were found to be negative for the mutation, a variant allele frequency (VAF) greater than 1% was considered positive.

### 2.4 Statistical analyses

Statistical analyses with a two‐tailed unpaired *t‐*test and non‐linear regression were performed with Prism software (version 9; GraphPad Software, Inc.). The Mann–Whitney U test and Fisher's exact test were performed using R, version 3.4.2. *p* values less than 0.05 were considered statistically significant.

## 3 RESULTS

### 3.1 Comparison of laboratory data in peripheral blood between CNSL and infectious/demyelinating diseases

**FIGURE 1:**
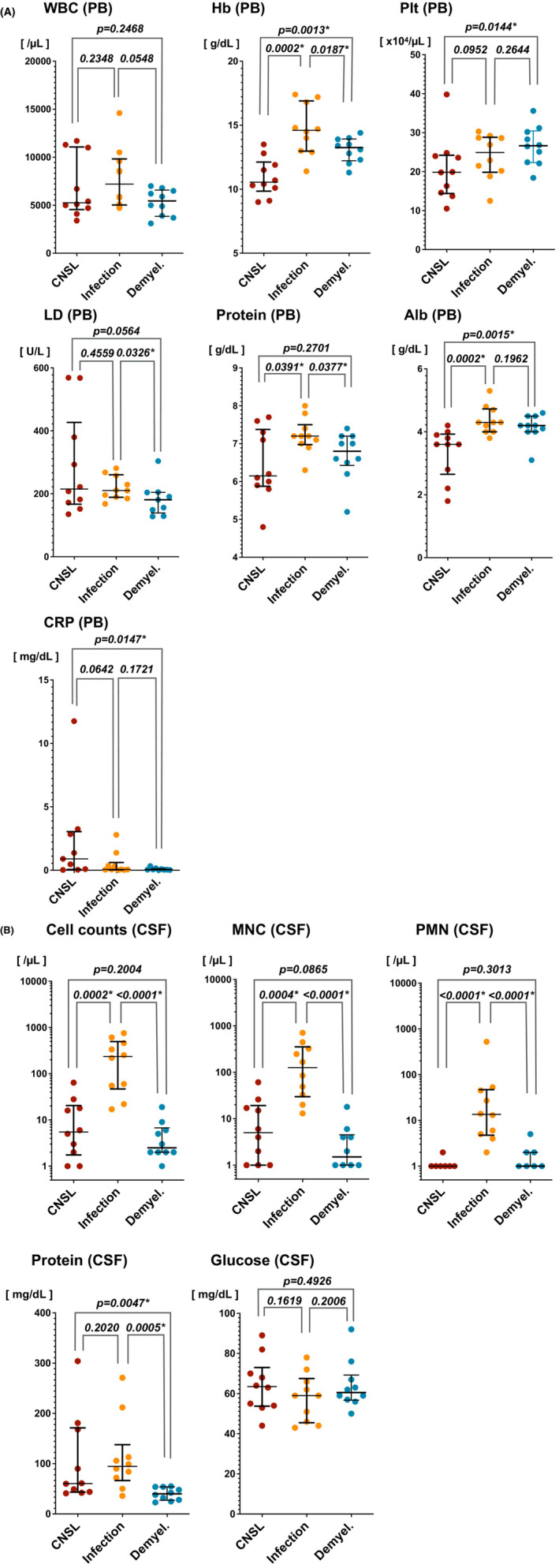
Comparison of laboratory data in peripheral blood (PB) and cerebrospinal fluid (CSF) in CNSL, CNS infectious diseases, and demyelinating diseases. (A) Laboratory data in peripheral blood and (B) CSF. Scatter plots with medians and interquartile range are shown. The asterisks (*) indicate significant differences. Alb, albumin; Hb, hemoglobin; LD, lactate dehydrogenase; Plt, platelets; WBC, white blood cells.

In the clinical setting, peripheral blood and CSF examinations are usually performed primarily for the differentiation of infectious, demyelinating and neoplastic diseases of the CNS. To identify differences in characteristics between CNSL and infectious/demyelinating diseases, values of peripheral blood cell counts and key biochemical data were compared (Table 1; Figure 1A). Hemoglobin (Hb) level was found to be significantly lower in CNSL than in infectious and demyelinating diseases (*p* = 0.0002 and *p* = 0.0013, respectively), and although platelet counts were significantly lower in CNSL than demyelinating diseases (*p* = 0.0144), no significant difference was identified between infectious diseases and CNSL (*p* = 0.0952). Serum lactate dehydrogenase (LD) levels tended to be higher in CNSL than demyelinating diseases (*p = 0.0564*), although the difference was not significant. Total protein was significantly higher in infectious diseases than in CNSL and demyelinating diseases (*p = 0.0391* and *p = 0.0377*, respectively), although serum albumin (Alb) was significantly lower in CNSL than in infectious and demyelinating diseases (*p = 0.0002* and *p = 0.0015*, respectively). CRP was significantly higher in CNSL than in demyelinating diseases (*p = 0.0147*), and showed a higher tendency in CNSL than in infectious diseases (*p = 0.0642*). These results suggest that lower Hb, lower Alb, and higher CRP in peripheral blood, in addition to clinical symptoms and imaging findings, including MRI, might be suggestive features of CNSL when differentiating CNSL from infectious/demyelinating diseases. Further, LD values tended to be lower in demyelinating diseases than in CNSL and infectious diseases.

### 3.2 Comparison of laboratory data in cerebrospinal fluid in CNSL and infectious/demyelinating diseases

Examination of laboratory data using CSF showed that total cell counts were significantly higher in infectious diseases than in CNSL and demyelinating diseases (*p* = 0.0002 and *p* ≤ 0.0001, respectively). Similar results were confirmed for both mononuclear cells (MNC: *p* = 0.0004 and *p* < 0.0001, respectively) and polymorphonuclear cells (PMN: *p* < 0.0001 and *p* < 0.0001, respectively). CSF protein levels were significantly lower in demyelinating diseases than in CNSL and infectious diseases (*p = 0.0047* and *p = 0.0005*, respectively). No significant differences were identified in spinal fluid glucose levels. The results suggest that elevated CSF cell counts are not a feature of CNSL but rather a finding in infectious diseases. The results also suggest that it is difficult to distinguish CNSL from infection based on CSF protein and glucose levels.

### 3.3 CSF‐cfDNA concentration in CNSL and infectious/demyelinating diseases

**FIGURE 2:**
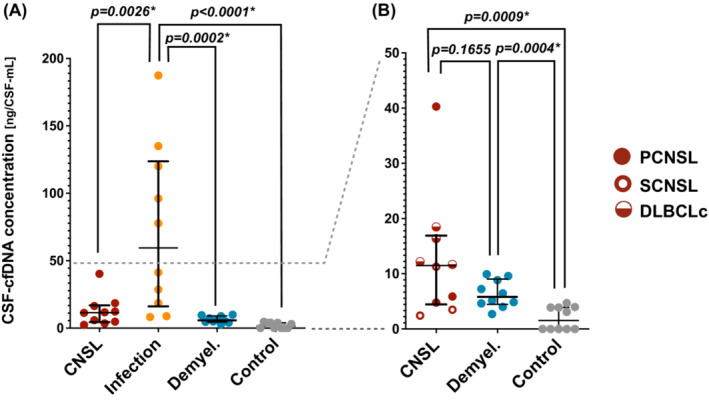
Comparison of CSF‐cfDNA concentration in CNSL, cerebrospinal infectious diseases and demyelinating diseases. (A) Scatter plots of CSF‐cfDNA concentration in CNSL, infectious diseases, demyelinating diseases and CSF from volunteers without cerebrospinal diseases indicating the median and interquartile range. Note that the concentration in infectious diseases was significantly higher than that in the other conditions. (B) CSF‐cfDNA concentration in the three groups other than the infectious diseases group is depicted for easy comparison. The three disease conditions of CNSL are indicated by different symbols in the graph. CSF‐cfDNA concentrations were significantly higher in CNSL and demyelinating disease groups than in the volunteer group. The asterisks (*) indicate significant values.

CSF‐cfDNA concentrations were compared using CSF obtained from patients with CNSL, infectious diseases, demyelinating diseases, and control participants without CNS diseases (Figure 2A,B). CSF‐cfDNA levels were found to be significantly higher in infectious diseases than in CNSL and demyelinating diseases (*p* = 0.0026 and *p* = 0.0002, respectively). CSF‐cfDNA levels in CNSL were significantly higher than those in controls (*p* = 0.0009), although with no significant difference versus demyelinating diseases (*p* = 0.1655) (Figure 2B). In terms of cfDNA concentrations in the three CNSL disease types (PCNSL, SCNSL, and DLBCLc) (Figure 2B), although the cfDNA concentration tended to be higher in DLBCLc compared to PCNSL/SCNSL, further study is surely warranted due to the small number of cases in this cohort. These results suggest that high CSF‐cfDNA levels are not a hallmark of CNSL, but rather might be important for differentiating infectious diseases.

### 3.4 Mutational status of *MYD88* ^L265P^ and *CD79B* ^Y196^ using CSF‐cfDNA ddPCR

**FIGURE 3:**
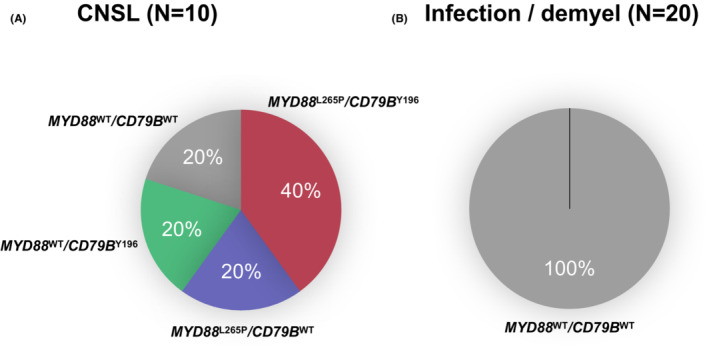
Detection of genetic mutations of *MYD88* ^L265P^ and *CD79B* ^Y196^ using CSF‐cfDNA in CNSL and cerebrospinal infectious/demyelinating diseases. (A) Mutation status in CNSL is shown in the pie chart. Note that 80% of the CNSL patients analyzed demonstrated one of the mutations, as assessed using ddPCR. (B) Mutation status in cerebrospinal infectious/demyelinating diseases. None of the patients had detectable genetic mutations.

The results of mutation analysis by ddPCR using CSF‐cfDNA are shown in Table 2 and Figure 3. *MYD88* ^L265P^ and *CD79B* ^Y196^ were each detected in six (60%) CNSL cases. Mutual existence of *MYD88* ^L265P^ and *CD79B* ^Y196^ was identified in three of three PCNSL cases (100%), none of the three SCNSL cases (0%), and one of the DLBCLc (25%) cases, respectively. The mutations were not observed in infectious and demyelinating diseases. These results indicate that the sensitivity and specificity of positive tests for *MYD88* ^L265P^ and *CD79B* ^Y196^ in CNSL were 80% and 100%, respectively. Moreover, these results suggest that detection of *MYD88* ^L265P^ and *CD79B* ^Y196^ by genetic analysis using CSF‐cfDNA might provide useful information for differentiating CNSL from infectious/demyelinating diseases.

In four cases from whom biopsy specimens of brain lesions were available, ddPCR analysis was performed using genomic DNA extracted from FFPE (Table 2). In UPN (unique patients number) #2, the same *MYD88* ^L265P^ mutation detected by CSF‐cfDNA was confirmed by ddPCR but in UPN #6, the *MYD88* ^L265P^ mutation was not detected by CSF‐cfDNA but was detected by ddPCR analysis of the FFPE sample. In the three cases (UPN # 2, 6, and 10) in which *CD79B* ^Y196^ was detected in CSF‐cfDNA, these mutations could not be detected by mutation analysis using FFPE DNA. These results suggest that sufficient consideration should be given to the possibility of regional heterogeneity at each collection site in the analysis of genetic mutations using brain biopsy specimens.

### 3.5 Comparison of detection timings using genetic mutations versus cytological and/or pathological abnormalities

**FIGURE 4:**
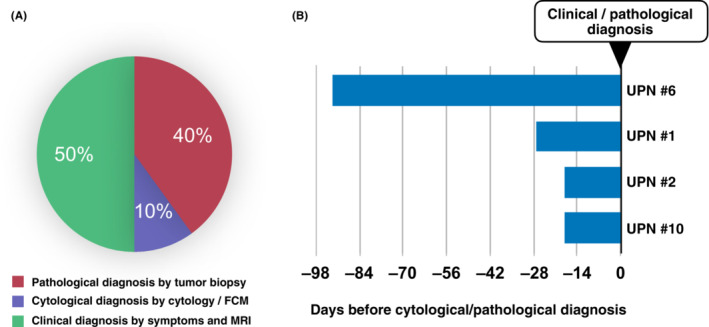
Detection of genetic mutations using CSF‐cfDNA prior to obtaining pathological and/or cytological diagnosis in CNSL patients. (A) Strategies of obtaining diagnosis of CNSL (*N* = 10). (B) Early detection of genetic mutations in CNSL prior to diagnosis by conventional strategies. In cases in which the diagnosis of CNSL was made using brain biopsy, cytology in cerebrospinal fluid, or detection of tumor cells by FCM, the date of collection of these specimens was set as Day 0. The date of collection of CSF specimens in which *MYD88* ^L265P^ and/or *CD79B* ^Y196^ were detected, which was before the pathological/cytological diagnosis was made, is shown. In the four available cases, it was possible to detect mutations of *MYD88* ^L265P^ and/or *CD79B* ^Y196^ in CSF samples obtained before the definitive diagnosis by conventional strategies.

We retrospectively examined whether mutation analysis using CSF‐cfDNA is useful for the early detection of CNSL. In the clinical setting, CSF examination is performed soon after the first suspicion of neoplastic, inflammatory, or demyelinating CNS diseases. A definitive diagnosis of CNSL is made by detection of tumor cells in CSF using cytology and FCM, as well as by biopsy of the tumor lesion. The strategies used for definitive diagnosis of the CNSLs in this study are shown in Table S1 and Figure 4A. In five cases (50%), the diagnosis was confirmed by brain biopsy or cytology, and in the other five cases (50%), CNS invasion was clinically determined by histological evaluation of the primary extraneural tumor, CNS symptoms, and MRI findings. Although Case 1 was finally diagnosed by cytology of CSF cells and FCM, the first CSF specimen (positive for *MYD88* ^L265P^ and *CD79B* ^Y196^) was not cytologically diagnostic in the early stages of disease onset (previously reported by Iriyama^4^). Four of the five cases (UPN #s 1, 2, 6, 9, and 10), in which CSF collection was performed prior to cellular and histological diagnosis, were mutation positive in CSF‐cfDNA analyses. The time from CSF collection to a definitive diagnosis in these cases is shown in Figure 4B. In these four cases, it is possible that the genetic mutations might have been detectable as early as 2–13 weeks before definitive diagnosis was obtained. In four of the five cases where the diagnosis could not be made by cyto‐histology, the mutations could be detected by CSF‐cfDNA. These data strongly suggest that detection of *MYD88* ^L265P^ and *CD79B* ^Y196^ mutations using CSF‐cfDNA has high sensitivity and specificity for diagnosing CNSL and might be a useful strategy for the early detection of CNSL.

## 4 DISCUSSION

In cases with suspected CNSL, CSF examination is a clinically important test performed in the early stages of the disease. Our data showed that elevation of CSF cell count is an important finding in infectious diseases but not in CNSL, and that a definitive diagnosis by cytology or FCM is quite difficult in the early stages of CNSL. We observed a significant decrease in blood Hb and Alb levels in CNSL compared to infectious and demyelinating diseases, which might reflect the systemic symptoms caused by malignant lymphoma. Our results also suggested that high LD levels might be useful for differentiating CNSL from demyelinating diseases but not from infectious diseases. Although it was difficult to conduct a more detailed analysis in this study since it focused on lymphomas with CNS involvement, these results might differ if the analysis is limited to PCNSL, and this is a subject for future study. Several reports have also evaluated the usefulness of soluble interleukin‐2 receptor (sIL‐2R), IL‐6, and IL‐10 values in CSF^33^ but we did not assess these parameters since this study was a retrospective analysis in a clinical setting, and because of the limitations of the insurance system in Japan. Analysis of these values together might be an issue for future discussion.

Surprisingly, CSF‐cfDNA concentrations were not particularly high in CNSL cases. The high CSF‐cfDNA concentrations in infectious diseases is similar to the trend in cell counts, suggesting that the cfDNA is derived from inflammatory cells in the spinal fluid. Previous analysis of peripheral blood cfDNA (PB‐cfDNA) confirmed that PB‐cfDNA concentrations are high particularly in DLBCL and IVLBCL as compared to malignant lymphomas,^18^ and the high concentrations have been suggested to reflect the disease status. Therefore, although it is difficult to distinguish CNSL from other diseases based on CSF‐cfDNA concentration alone, it might be possible to infer disease status by serial changes in its value over time. This is an issue for future study.

Mutation analysis using CSF‐cfDNA detected at least one of the mutations of *MYD88* ^L265P^ and/or *CD79B* ^Y196^ in 80% of the CNSL cases examined, as reported previously.^7^, ^8^, ^11^ However, since the previous studies have not yet accumulated enough information on mutation prevalence in diseases other than CNSL, it is unclear whether a positive result on mutation analysis is strongly suggestive of CNSL. In our analyses, no *MYD88* ^L265P^ and/or *CD79B* ^Y196^ mutations were detected in infectious or demyelinating diseases. Both the sensitivity and specificity of the results were very high, indicating that CNSL should be strongly suspected when these mutations are detected.

In our experiment, cases of SCNSL and systemic DLBCL (DLBCLc) were included in addition to PCNSL. Previous reports of genetic mutation analysis in CNSL have mostly been limited to PCNSL.^7^, ^8^, ^11^ In the present analysis, 5/7 patients (71%) were found to harbor *MYD88/CD79B* mutations in SCNSL and DLBCLc as well. Patients with these mutations are considered to have the MCD subtype of DLBCL, and the present results may reflect the fact that the MCD subtype is characterized by a predisposition to CNS invasion.

Another important finding of the present study was that *MYD88/CD79B* mutations could be detected in CNSL cases (e.g., Case 2) in which brain biopsy could not be performed. Positive findings on mutation analysis using liquid biopsy techniques in difficult‐to‐biopsy cases are highly suggestive of the presence of CNSL, and they are considered to be extremely useful clinically for ruling out inflammatory/demyelinating diseases. However, since histology is currently considered the gold standard for making a definitive diagnosis of lymphoma, further discussion is needed to determine whether detection of these mutations might contribute to making a definitive diagnosis. It is important to note that about 20% of the CNSL cases in this study were negative for these mutations, suggesting that a negative result does not rule out CNSL, and repeated analyses using conventional strategies is necessary when CNSL is clinically suspected.

Mutation analysis using CSF‐cfDNA and genomic DNA from FFPE specimens showed some divergence in results. It has been experienced in previous comprehensive genetic mutation analyses that some genetic mutations are detected only in one or the other sample.^18^ Possible reasons for this phenomenon have been discussed, including the influence of regional heterogeneity and the presence of lesions that are more fragile and more prone to gene leakage into the liquid. In the present study, it was also necessary to consider the sample size and tumor existing percentage in biopsy tissue, and furthermore, the DNA quality extracted from FFPE specimens and the sensitivity of ddPCR analysis. Since the present analysis is a retrospective analysis of a limited number of cases, it would be desirable to examine this issue in a prospective study in the future, setting standards for biopsy methods and specimen quality. In our analysis, no mutation‐positive cases were observed in infectious/demyelinating diseases, suggesting that the signal‐to‐noise ratio is preserved. Hence, mutation analysis using CSF‐cfDNA has potential utility not only for examining cases that cannot be biopsied, but also to more sensitively detect diseases than analysis using biopsy specimens. Further accumulation of cases is desirable for confirming our results.

The correlation between MRI imaging findings, location of the tumor, and the specific mutation is also of interest. In this context, another important question is whether the CSF‐cfDNA concentration differs depending on the location and size of the tumor in the CNS, and whether the tumor location affects the sensitivity of mutation detection. It appears that the size of the lesions on brain imaging does not necessarily determine the likelihood of positivity or negativity of the liquid biopsy results, as evidenced by the positive liquid biopsy results with smaller lesions in Cases 6 and 7, as compared to the larger lesions but negative liquid biopsy results in Cases 4 (SCNSL) and 9 (DLBCLc). However, since the number of cases in this study was limited, these are considerations for a future study.

Cases of IVLBCL of the CNS were excluded in this study. In IVLBCL, mutation analysis using plasma‐cfDNA has been reported to be highly sensitive,^18^, ^34^ and our limited experience suggests that plasma‐cfDNA might be more sensitive than CSF‐cfDNA for the detection of IVLBCL (^32^ and unpublished data). This phenomenon could be related to the fact that the CSF and intravascular space are partitioned by the blood–brain barrier. Further accumulation of cases is needed to determine the usefulness of plasma‐cfDNA and CSF‐cfDNA in diagnosing primary IVLBCL of the brain.

In conclusion, we confirmed that observation of *MYD88* ^L265P^ and *CD79B* ^Y196^ mutations in CSF‐cfDNA in cases of lymphomas with CNS involvement might be useful for distinguishing them from cerebral infectious/demyelinating diseases, even in cases where brain biopsy is difficult to perform. Since the present study was a retrospective study limited to cases with specimen preservation, the analysis was limited to a small number of cases. It is necessary to conduct a prospective study in the future to accumulate more cases and validate the results of this study. Further careful evaluation is needed to determine whether the presence of these genetic abnormalities, in addition to indirect findings such as clinical symptoms, laboratory findings, and imaging findings, can be used to diagnose CNSL.

## AUTHOR CONTRIBUTIONS

**Chisako Iriyama:** Conceptualization (equal); data curation (equal); formal analysis (equal); investigation (lead); methodology (equal); resources (equal); writing – original draft (equal); writing – review and editing (equal). **Kenichiro Murate:** Conceptualization (equal); formal analysis (equal); investigation (equal); methodology (equal); resources (equal); writing – original draft (equal); writing – review and editing (equal). **Sachiko Iba:** Data curation (equal); formal analysis (equal); investigation (equal); methodology (equal); resources (equal); validation (equal); visualization (equal). **Akinao Okamoto:** Methodology (supporting); writing – review and editing (supporting). **Naoe Goto:** Investigation (supporting); writing – review and editing (supporting). **Hideyuki Yamamoto:** Investigation (supporting); writing – review and editing (supporting). **Toshiharu Kato:** Investigation (supporting); writing – review and editing (supporting). **Keichiro Mihara:** Investigation (supporting); writing – review and editing (supporting). **Takahiko Miyama:** Investigation (supporting); writing – review and editing (supporting). **Keiko Hattori:** Investigation (supporting); methodology (supporting); writing – review and editing (supporting). **Ryoko Kajiya:** Investigation (supporting); methodology (supporting); writing – review and editing (supporting). **Masataka Okamoto:** Investigation (supporting); writing – review and editing (supporting). **Yasuaki Mizutani:** Investigation (supporting); writing – review and editing (supporting). **Seiji Yamada:** Investigation (supporting); writing – review and editing (supporting). **Tetsuya Tsukamoto:** Investigation (supporting); methodology (supporting); writing – review and editing (supporting). **Yuichi Hirose:** Methodology (supporting); project administration (supporting); writing – review and editing (supporting). **Tatsuro Mutoh:** Project administration (supporting); writing – review and editing (supporting). **Hirohisa Watanabe:** Conceptualization (equal); data curation (equal); methodology (equal); project administration (equal); resources (equal); supervision (equal); writing – original draft (equal); writing – review and editing (equal). **Akihiro Tomita:** Conceptualization (equal); data curation (equal); formal analysis (equal); funding acquisition (equal); investigation (equal); methodology (equal); project administration (equal); supervision (equal); writing – original draft (lead); writing – review and editing (lead).

## FUNDING INFORMATION

This work was supported in part by Grants‐in‐Aid from the Ministry of Education, Culture, Sports, Science and Technology, Japan (15K09473 and 21K08407 to A.T.).

## CONFLICT OF INTEREST STATEMENT

K.M.: Research funding: TAKARA BIO. Lecture fees: Janssen Pharmaceutical, Sanofi, and CSL Behring. T.M.: Research funding: The Smoking Research Foundation. Consulting fee: Nippon Zoki Pharmaceutical Co. Ltd. A.T.: Research funding: Novartis Pharma, Pfizer Japan, PPD, and Perseus Proteomics. Scholarship (incentive) endowments: Chugai Pharmaceutical, Ono Pharmaceutical, and Eisai. Lecture fees: Chugai Pharmaceutical, AstraZeneca, Ono Pharmaceutical, Eisai, Takeda Pharmaceutical, and Nippon Shinyaku.

## ETHICS STATEMENT

This study was approved by the institutional review board of Fujita Health University (approval numbers, HG18‐017, HG20‐055, HG21‐028, and HG22‐006). Informed consent was obtained from all cases. Registry and the Registration No. of the study/trial: N/A. Animal Studies: N/A.

## Supporting information


Data S1.

Table S1.

## Data Availability

The data that support the findings of this study are available from the corresponding author upon reasonable request.
