# Supplementary material for: Utility of cerebrospinal fluid liquid biopsy in distinguishing CNS lymphoma from cerebrospinal infectious/demyelinating diseases
Source: Cancer Med. 2023 Jul 27;12(16):16972–84. doi: 10.1002/cam4.6329 (PMC10501233; doi:10.1002/cam4.6329)
Supplement: Supplementary file 1 — Data S1. [file CAM4-12-16972-s001.pdf]

Supplementary information

Supplementary Table

Table S1 Patients' characteristics and diagnostic strategy for detection of CNS involvement of lymphoma

UPN	Disease category	Pathological diagnosis	Sex	Age	Strategies for diagnosis of CNS involvement	CSF			Time from CSF collection to cytological/pathological diagnosis [months]	Strategies for initial diagnosis at first onset in SCNSL and DLBCLc	Stage	IPI	CNS-IPI
						Cytlogy	FCM	ddPCR					
**1**	**PCNSL**	B-NHL	M	61	CSF cytology, FCM using MNCs in CSF*	(-)	(-)	(+)	27	N.A.	I	4	N.A.
**2**	**PCNSL**	DLBCL	F	73	Brain biopsy	(-)	N.A.	(+)	18	N.A.	I	3	N.A.
**3**	**PCNSL**	N.A.	F	65	Clinical symptoms, MRI**	(-)	N.A.	(+)	N.A.	N.A.	I	2	N.A.
**4**	**SCNSL**	DLBCL	F	72	Clinical symptoms, MRI	(-)	N.A.	(-)	N.A.	Inguinal LN biopsy	IV	3	3
**5**	**SCNSL**	DLBCL	M	73	Clinical symptoms, MRI	(-)	(-)	(+)	N.A.	Duodenum tumor biopsy	IV	3	4
**6**	**SCNSL**	DLBCL	F	53	Brain biopsy	(-)	(-)	(+)	93	Breast tumor biopsy	IV	3	3
**7**	**DLBCLc**	DLBCL	M	66	Clinical symptoms, MRI	(-)	(-)	(+)	N.A.	Cervical LN biopsy	IV	5	6
**8**	**DLBCLc**	DLBCL	M	70	Clinical symptoms, MRI	(-)	(-)	(+)	N.A.	Retroperitoneal LN biopsy	IV	5	6
**9**	**DLBCLc**	DLBCL	M	76	Brain biopsy	(-)	N.A.	(-)	24	Brain biopsy	IV	5	6
**10**	**DLBCLc**	DLBCL	F	63	Brain biopsy	(-)	N.A.	(+)	18	Brain biopsy	IV	4	4

CNSL: central nerves system lymphoma, PCNSL: primary CNSL, SCNSL: secondary CNSL, DLBCLc: diffuse large B cell lymphoma with CNS involvement, B-NHL; B-cell non-Hodgkin lymphoma, (+); detectable, (-) un-detectable, N.A.; not applicable, IPI; international prognostic index, CNS-IPI; CNS-international prognostic index. *Analysis using specimens approximately 1 month after initial CSF collection that was mutation-positive, **vitreous biopsy was performed, but pathological diagnosis was not obtained.

Supplementary Figure

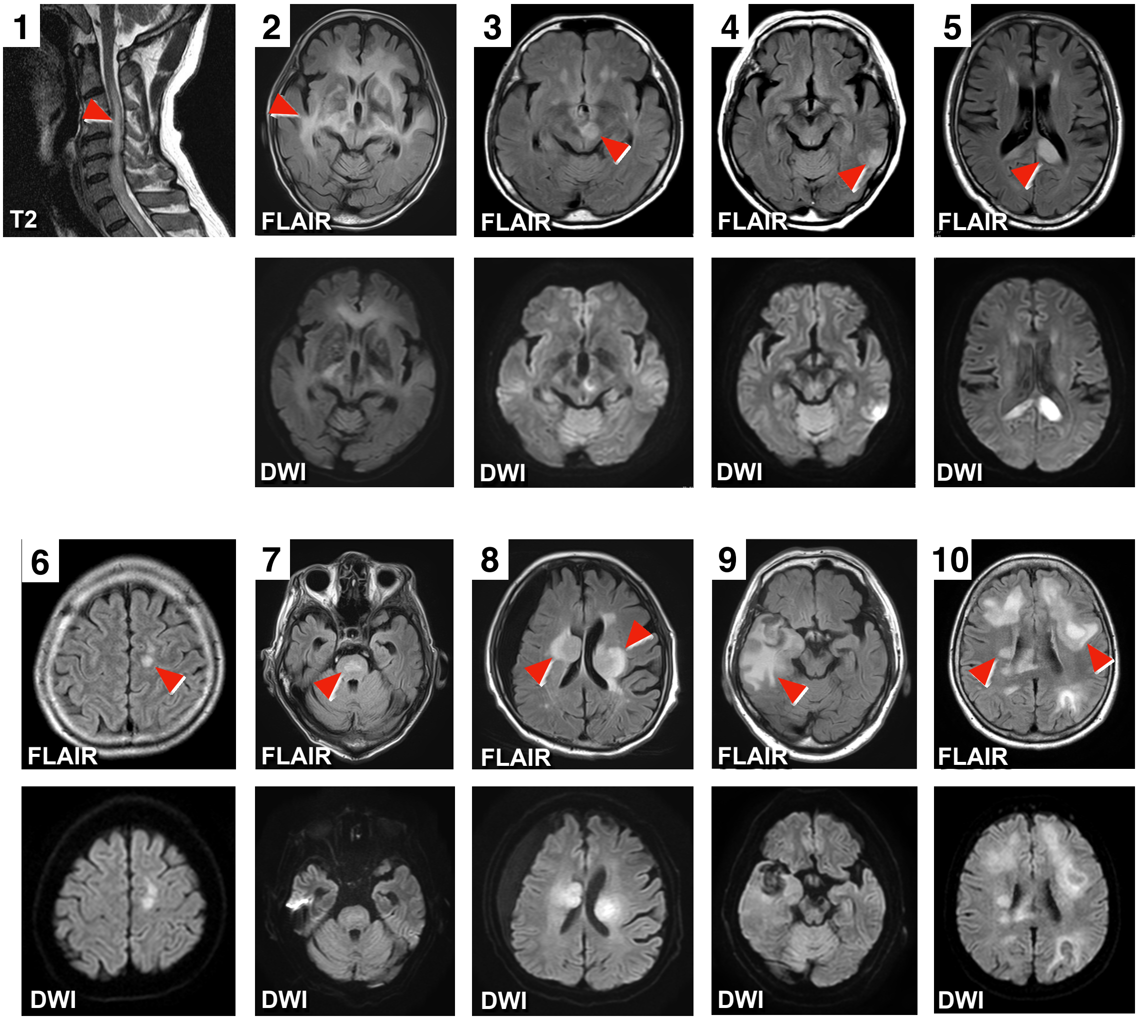

Figure S1 Magnetic resonance imaging (MRI) findings in each CNSL case in this cohort

The numbers listed correspond to the case numbers in Table 2. CNSL usually shows higher signal intensity compared to white matter on T2-weighted images (T2), and lower signal intensity compared to the surrounding area of edema, with a uniform strong enhancing effect and high signal intensity on diffusion-weighted images (DWI) in most of the patients. The red arrows indicate areas suggestive of infiltration of lymphoma cells. Brain MR examinations were performed with a 3-T MR system (Vantage Galan 3T / ZGO, Canon Medical Systems, Otawara, Japan) using a 32-channel head coil (32ch Head SPEEDER, Canon Medical Systems). FLAIR: fluid-attenuated inversion recovery.
